# Hypoglossal Nerve Neuropathies—Analysis of Causes and Anatomical Background

**DOI:** 10.3390/biomedicines12040864

**Published:** 2024-04-14

**Authors:** Andrzej Węgiel, Nicol Zielinska, Mariola Głowacka, Łukasz Olewnik

**Affiliations:** 1 Department of Anatomical Dissection and Donation, Medical University of Lodz, 90-647 Lodz, Poland; andrzej.wegiel@stud.umed.lodz.pl (A.W.); nicol.zielinska@stud.umed.lodz.pl (N.Z.); 2 Nursing Department, Masovian Academy in Płock, 09-402 Płock, Poland; m.glowacka@mazowiecka.edu.pl; 3 Department of Clinical Anatomy, Masovian Academy in Płock, 09-402 Płock, Poland

**Keywords:** compression, hypoglossal nerve, iatrogenic, internal carotid artery, neuropathy, palsy, paralysis

## Abstract

The hypoglossal nerve is the last, and often neglected, cranial nerve. It is mainly responsible for motor innervation of the tongue and therefore the process of chewing and articulation. However, tumors, aneurysms, dissections, trauma, and various iatrogenic factors such as complications after surgeries, radiotherapy, or airway management can result in dysfunction. Correct differential diagnosis and suitable treatment require a thorough knowledge of the anatomical background of the region. This review presents the broad spectrum of hypoglossal neuropathies, paying particular attention to these with a compressive background. As many of these etiologies are not common and can be easily overlooked without prior preparation, it is important to have a comprehensive understanding of the special relations and characteristic traits of these medical conditions, as well as the most common concomitant disorders and morphological traits, influencing the clinical image. Due to the diverse etiology of hypoglossal neuropathies, specialists from many different medical branches might expect to encounter patients presenting such symptoms.

## 1. Introduction

### 1.1. Anatomy

The hypoglossal nerve (HN) is the twelfth cranial nerve. Its rootlets arise from the medulla oblongata medially in the ventrolateral sulcus and descend laterally behind the vertebral artery (VA). The rootlets usually form two bundles, which consecutively run through the dura matter and arachnoid in the hypoglossal canal inside the occipital bone. They then emerge from the canal, fuse together, and travel further medially as a single nerve to the internal carotid artery (ICA), internal jugular vein (IJG), and the ninth to the eleventh cranial nerves [1,2,3]. The HN becomes fused with the inferior vagal ganglion by the connective tissue exchanging numerous small nerve filaments. As the HN descends, it heads into the space between the internal ICA and the IJG. At this point, it receives the efferent fibers from the first cervical spinal nerve (C1), which later supplies the geniohyoid and thyrohyoid muscles. At the level of the atlas, it receives the sympathetic fibers from the superior cervical ganglion [3].

When it reaches the level of the angle of the mandible, it loops around the root of the occipital artery (OA) lying inferior to the posterior belly of the digastric muscle. At the level of the hyoid bone, the nerve takes a turn and starts heading anteriorly. Next, it crosses the lingual artery and moves forward along to the hypoglosus muscle in the submandibular area towards the extrinsic and intrinsic tongue muscles. At the end of its course, it penetrates the genioglossus muscle and reaches the tip of the tongue [2].

The first group of branches of the HN are the meningeal branches, which separate from the nerve within the hypoglossal canal. The second branch is the descending branch, which is given off when the HN curves around the OA. The third and fourth branches are nerves supplying the thyrohyoid and geniohyoid muscles. They originate near the posterior border of the hyoglossus muscles. The last group of branches are those reaching the muscles of the tongue (Table 1) [4].

**Figure 1:**
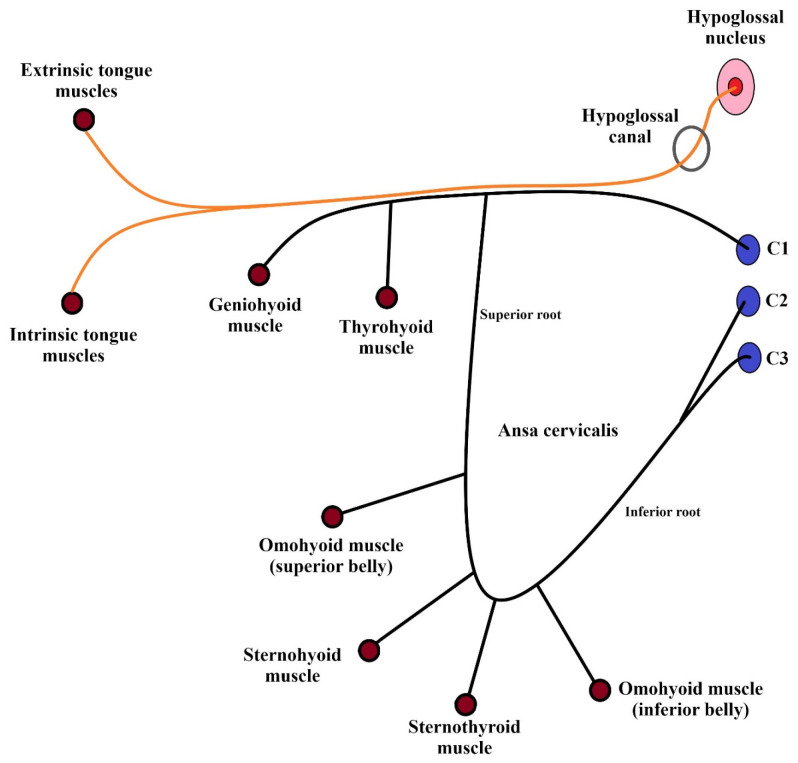
Schematic representation of the HN and the ansa cervicalis branches.

The HN supplies two groups of muscles: the intrinsic tongue muscles and the extrinsic tongue muscles. In addition, it carries fibers from C1 supplying the geniohyoid and the thyrohyoid muscles and C1 fibers that innervate the infrahyoid muscles through the ansa cervicalis. The only tongue muscle not supplied by the HN is the palatoglossus, which receives its innervation from the vagus nerve [1]. The tongue muscles are innervated only ipsilaterally (Figure 1) [3].

The HN receives blood supply consecutively from the VA, the ascending pharyngeal artery, the OA, the external carotid artery (ECA), and again from the ascending phalangeal artery near the bifurcation of the common carotid artery. Near the tongue and within it, blood is supplied by the lingual artery [5,6,7].

**Figure 2:**
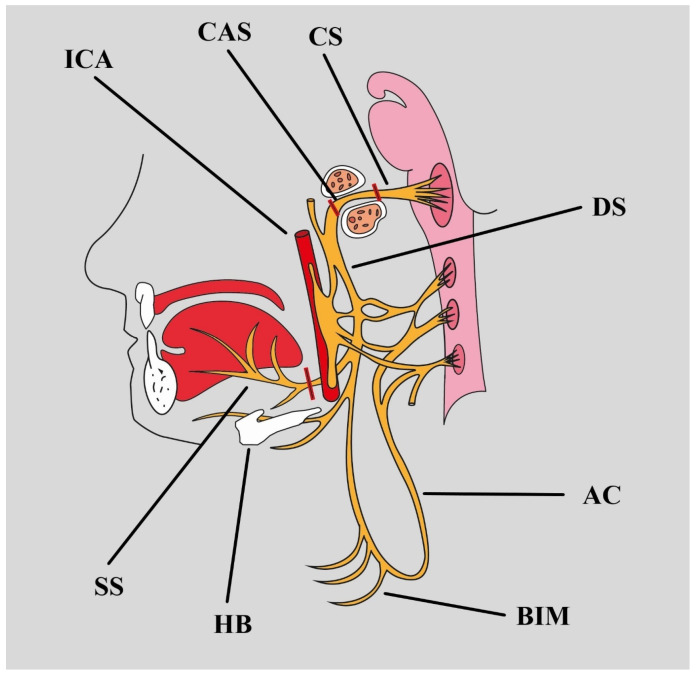
Segments of the HN: ICA internal carotid artery, CAS canalar segment, CS cisternal segment, DS descending segment, AC ansa cervicalis, BIM branches to infrahyoid muscles, HB hyoid bone, SS sublingual segment.

The HN can be divided into five segments: cisternal, canalar, descending, horizontal, and ascending. Horizontal and ascending segments can be called together as sublingual (Table 2) (Figure 2) [8].

### 1.2. Anatomical Variants

The nerve is characterized by diverse morphological variability along its length. The number of rootlets varies from three to fifteen. They later fuse into two or three trunks [6]. An aberrant course can sometimes pose a surgical risk of surgical complications, such as crossing perpendicular and superficially to the IJG [9,10]. The number of terminal branches supplying each of the muscles also significantly varies; for example, the terminal branch to the thyroid muscle can be duplicated [4,11]. In addition, sternocleidomastoid, stylohyoid, digastric, or mylohyoid muscles can also receive innervation from the HN [4,12].

### 1.3. Clinical Basics

Patients with an isolated HN pathology can present with dysphagia, dysarthria, and tongue muscular atrophy. They also demonstrate such tongue dysfunctions as deviation, dysarthria, weakness, or fasciculation. In cases of bilateral palsy, it may occur accumulation of saliva in the oral cavity [13]. The level of the nerve injury can be determined based only on observation of symptoms. Supranuclear damage manifests contralateral to the site of the lesion, hence tongue deviates away from the side of injury but it does not exhibit visible atrophy or fasciculation. Nuclear or infranuclear damage results in dysfunction appearing ipsilaterally to the affected side [14]. Difficulties in swallowing indicate infrahyoid muscle involvement, which is innervated by branches from C1 to C3; this suggests that the lesion is present peripherally, i.e., distally to the insertion of C1 with the HN [2].

Half of all HN impairments were reported to be of tumor origin; however, 3–15% were idiopathic, i.e., without any obvious lesion or cause, even with the use of advanced diagnostic tools [13,15]. Idiopathic cases are mostly seen among younger patients [16]. They are typically expected to resolve spontaneously without any treatment, similar to Bell’s palsy but longer-term cases have also been described [17,18]. However, isolated cases of HN palsy in this group should be taken seriously as they might represent the first sign of underlying malignancy [19].

A diagnosis of HN palsy of unknown origin should include a radiological examination of the brain, carotid, and chest regions in order to rule out vascular pathologies and oncologic and inflammatory tumors. In addition, tests targeted at autoimmunologic diseases and infectious agents (mostly EBV, CMV, and HSV) should be performed. An MRI scan every couple of years may be considered for visualizing a possible demyelinating etiology [20].

Although this article is devoted mainly to describing a broad spectrum of pathologies possibly affecting the HN, it is important to remember that in many instances, the HN palsy is not an isolated deficit. A study by Stino et al. [13] identified isolated involvement of the HN in 49% of cases. Therefore, when the pathologic process is active in its proximity, it is reasonable to expect various degrees of symptoms also from the other cranial nerves (most commonly V, VI, VII, X) or the surrounding tissues. Their presence may blur the clinical image and delay the final diagnosis [13].

### 1.4. Materials and Methods

We conducted a search in the PubMed database using the terms “hypoglossal nerve compression”, “hypoglossal nerve neuropathy”, “hypoglossal nerve paralysis”, and “hypoglossal nerve palsy”. Based on our findings, we created the main sections of the article and continued the search for original articles for each section accordingly. We also analyzed reference lists of the gathered articles. Studies dating back several decades were included if the observations presented in them are still relevant. Paragraphs with clinical suggestions were based exclusively on the latest research. Clinical cases were included if they provided valuable information and larger sample studies were not available.

## 2. Vessel-Induced Neuropathies

The most diverse group of factors causing HN palsies are those associated with vascular problems. A cadaveric study by Bademci et al. [21] described a triple cross of the HN potential injury points. The first crossing is of the anterior medullary segment of the VA with the nerve roots; its importance is associated with pathologies of the VA and posterior inferior cerebellar artery (PICA). Next, the ansa cervicalis crosses the OA (75% of specimens), sternocleidomastoid artery and vein (15%), or ECA (10%) [21]. This is an important consideration when avoiding complications of the surgeries in this region, especially with those related to the ICA. The final cross is located in the submandibular triangle between the sublingual part of the HN and the vena commitans of the HN; this may play a role in tongue flap surgeries [21].

### 2.1. Vertebral Artery

The dilation of the VA is believed to potentially trigger HN neuropathy by compressing HN rootles. This condition may be limited solely to the VA, but it may also occur together with ectasia of the basilar artery [22]. In such cases, multiple neurological deficits involving several cranial nerves are observed. They typically extend beyond unilateral symptoms of isolated HN palsy.

Compression might not only be caused by an anomalous shape of the VA but also by its altered course. A dolichoectatic VA with an anomalous course was seen both in adults and juveniles. The compression of the HN occurs at the level where its rootlets emerge from the medulla oblongata, resulting in neuropathy [23,24,25]. A vertebral or basilar dolichoectasia was found in 6.48% of human cadavers [26].

**Figure 3:**
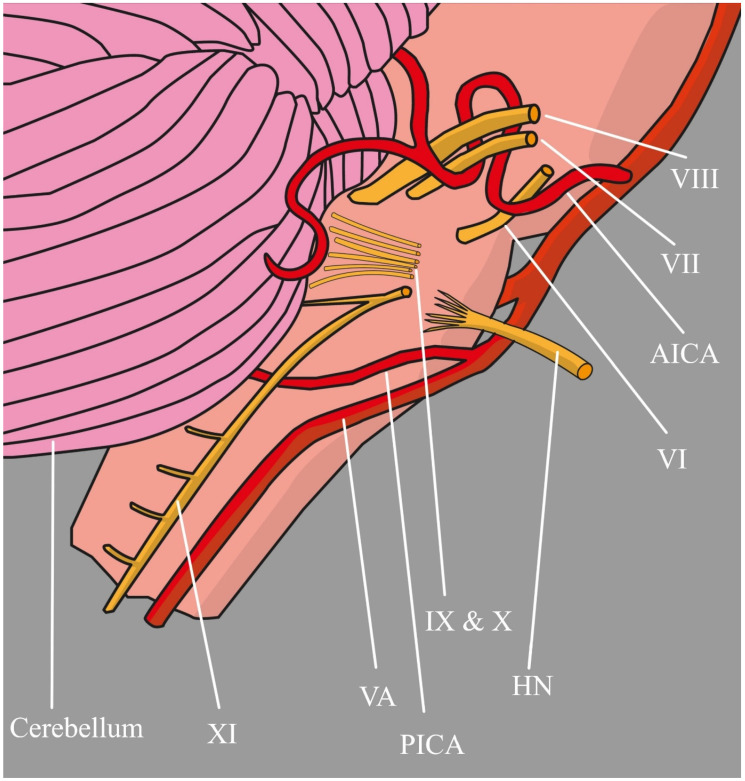
Relations between the HN and the adjacent arteries: XI accessory nerve, VA vertebral artery, PICA posterior inferior cerebellar artery, IX and X rootlets of vagus and glossopharyngeal nerves, HN hypoglossal nerve, VI abducens nerve, AICA anterior inferior cerebellar artery, VII facial nerve, VIII vestibulocochlear nerve.

Mass effect and compression by vascular anomalies are the most obvious mechanisms leading to nerve degeneration and hypoxia. It can also be caused by the rhythmic action of the vessel wall adjacent to the nerve. Its repetitive pulsatile movement might be sufficient to provoke the onset of symptoms without any visible pathology in the vessel itself: just the proximity of the artery to the nerve root exit zone. At its origins from the medulla oblongata, the nerve may be particularly susceptible to such pressure due to its transition between the central and peripheral myelin [27,28]. Similar compression was described by Rollnik et al. [29] but the etiologic factor was a kinking of the VA, while Giuffrida et al. [30] report pathology of the VA coexisting with an anomaly in PICA and hypertension (Figure 3).

In cases of compression by vascular anomalies, the evolution of symptoms may take a slow, progressive course [31]. A combination of MRI and magnetic resonance angiography is recommended as the best diagnostic option to visualize the altered course of the vessels and the precise anatomical relations between the adjacent structures [24].

### 2.2. Carotid Arteries

The relations between the HN and the ECA can be classified into three types [32,33]. Type I—the HN crossing the ECA inferior to the origin of the OA. Type II—the HN crossing the ECA at the level of origin of the OA. Type III—the HN crosses the ECA superior to the level of origin of the OA. In Type III, the HN is embraced by the loop of the OA. This variant has been proposed as a predisposing factor to nerve injury acting via prolonged external exertion of additional pressure and traction of the nerve [32]. Type II has a lower possibility of compression compared to Type III, but still, it cannot be completely ruled out. The other arteries that were seen looping around the HN were sternocleidomastoid branches coming from the OA or a singular sternocleidomastoid branch originating from the ECA. A low origin of the OA increases the risk of occurrence of Type III [32].

The lateral position of the ECA may also be another factor promoting impingement of the nerve. This variant was found in 4.9% of angiograms, more often on the right side [34]. Ueda et al. [34] report it to co-occur with an abnormally high position of the bifurcation of the common carotid artery. This high bifurcation might be considered a risk to the HN, especially in situations when it is accompanied by carotid arteries tortuosity and dolichoectasis [35].

Dokdod et al. [36] observed the abnormal loop of the ICA embracing and compressing the sublingual part of the HN. The presented ICA also had additional abnormalities of its wall contributing to a pathological condition. In such cases, antithrombotic drugs should be considered in order to prevent complications related to dissection and vascular complications.

Aneurysms are less frequent in the cervical portion of the ICA than in the external or common carotid arteries [37]. Among 1118 cases of peripheral artery aneurysm, four were associated with the ICA [38]. The most characteristic indications of an ICA aneurysm are a tender mass located in the neck and neurologic symptoms. When examining the mass, a pulsatile movement may indicate a vascular origin, but its absence does not exclude it. Other possible symptoms include headache, otorrhagia, epistaxis, and tinnitus. The frontal or occipito-parietal headache was also noticed in cases of idiopathic HN palsies lacking arterial dissections or aneurysms [39]. When there is an absence of central neurologic findings, direct cranial nerve involvement should be suspected rather than cerebral ischemia. Nevertheless, cases with manifestation limited only to the cranial nerves are considered extremely rare entities [40,41]. Aneurysms may be present in the company of other changes in the vessels such as tortuosity, distortion, and atherosclerosis [37].

The petrous segment of the ICA starts as the artery enters the base of the skull, in the petrous part of the temporal bone [42]. Although aneurysms in this segment are mostly asymptomatic, Cano-Duran et al. report a case of HN paralysis resulting from ICA dilation in its petrous part [40]. However, this should not be considered as its typical presentation.

An extracranial internal carotid aneurysm occurs in only 1% of patients with aneurysmal disease; within this group, 6–12% of cases demonstrate a bilateral variant [43,44]. Due to the high risk of embolism and secondary cerebral ischemia, surgical treatment is recommended both using intravascular methods and open resections with additional grafting [43].

### 2.3. Dissection

ICA dissections are the underlying cause of 5% to 22% of strokes in patients below 45 years of age, but its incidence peaks in the fifth decade of life. The annual incidence in the general population varies from between 2.5 and 3 persons per 100,000 [45]. The pseudoaneurysm is a serious complication of the injury to the arterial walls and in 12% to 17% of patients, it occurs within five years after the initial damage [46,47]. The damage to the nerve develops in two basic ways: direct compression or ischemia secondary to ligation or avulsion of the vasa nervorum [48]. Occlusive disease of the ICA can be caused by spasms, thrombosis, aneurysm, mural fibrosis, contusion, and, most frequently, intimal tear [49,50].

In rare occasions, HN palsy at the level of the cisternal segment may occur as a result of dissection of the VA. Dissections in general can be divided into subintimal and subadventitial types. In the former, the dissections typically obstruct the arterial lumen or cause thrombus that block it. In the latter, the pathologic changes are mainly directed beyond the arterial wall and can have a potential impact on adjacent structures, either by direct pressure or bleeding to the surrounding tissues [51]. The dissection may have its onset in the extracranial part and progress distally. Garnier et al. [52] report this to be present in 38% of cases. It is important to note that intracranial extension can be associated with subarachnoid hemorrhage [53]. Yet, the dissection does not necessarily reach the cisternal segment of the HN to trigger its palsy, as it has been reported to occur following distal embolization from the extracranial part of the VA. This also caused ischemia of the vasa nervorum [54]. The HN palsies resulting from dissections are considered reversible [53].

An additional factor contributing to nerve paresis may be the kinking of the artery, which can occur following dissection of the ICA with secondary intramural hematoma [55]. Such dissection was also reported to occur as a complication of trauma. There are four basic mechanisms of craniocervical injury that can end up as an ICA lesion: direct neck blow (50% of cases), stretching of the ICA over C1 vertebra with lateral neck flexion, blunt oral trauma, and intrapetrous thrombosis as a result of skull base fracture [56,57].

The onset of symptoms may be delayed for months after an accident. It has been proposed that this may occur through initially progressive, and then acute expansion of the ICA [57]. Connective tissue disorders such as Marfan’s syndrome, Ehlers–Danlos Type IV, and fibromuscular dysplasia may be predisposing factors to the dissection, as well as arterial tortuosity—a manifestation of vascular wall abnormality [53,58]. Saba et al. report that of three tortuous shapes of ICA, viz. kinking, coiling, and elongation, the first two were significantly associated with the presence of dissection [59]. The co-occurrence of ICA dissection and HN palsy was estimated as 5% [60].

The first symptoms that may indicate spontaneous infraclinoidal dissection of ICA include unilateral temporal headache, neck pain, bruises, visual symptoms, and cerebral deficits. The presence of oculosympathetic impairment in the form of partial Horner’s syndrome might be the result of damage to the sympathetic fibers looping around the ICA [41,47]. Horner’s syndrome may concern about 20% of patients with ICA dissection [61]. Due to the abundance of nerve plexuses supporting the vessels, the pain may be located distantly from the actual lesion, although it is mainly caused by the tearing of the vessel [41,47].

Diagnosis can be achieved without invasive techniques such as conventional angiography, for which the estimated risk of stroke ranges from 0.5% to 1% [45]. Magnetic resonance angiography provides a good insight into the vascular lumen while T1-weighted and T2-weighted MRIs allow detailed evaluation of the arterial wall. The most typical finding in carotid dissection in MRI examination is an intramural hematoma [62]. Another recommended diagnostic tool is CT angiography with rapid contrast bolus infusion. This method yields similar results to magnetic resonance techniques for diagnosing vertebral and carotid artery dissections; however, it is usually preferred in traumatic or emergency cases [63].

Although ultrasound is not the most accurate diagnostic option for suspected dissection, it does allow fast noninvasive imaging. It is particularly useful in cases with high levels or complete occlusions of arterial lumen and secondary ischemia; in such cases, the sensitivity of color Doppler is 95–96%, compared to 71% when the ischemia is not present [45].

### 2.4. Persistent Vessels

Persistent vessels are remnants of the primitive embryonic circulation that did not undergo expected involution. The presence of vascular pathology may be associated with enlargement of the hypoglossal canal. Shiozawa et al. [64] describe a case of an emissary vein emerging from the jugular vein and connecting with the IJG, which compressed the HN inside the hypoglossal canal and caused the dilatation of the outer opening of the canal. A similar pathology inside the hypoglossal canal can also be related to the persistent hypoglossal artery; such compression might be the effect of both segmental enlargement of the artery, or a result of its progressing calcification due to atherosclerotic changes [65,66]. The persistent hypoglossal artery was found to be associated with an increased risk of intracranial aneurysm [67].

Focal enlargement was also reported to occur secondary to aneurysms in the stump of the persistent hypoglossal artery, located just outside the skull base [68]. Dilation of the hypoglossal canal with preserved cortical margins is considered a normal observation in the presence of a persistent hypoglossal artery [69,70].

### 2.5. Dural Arteriovenous Fistula

Dural arteriovenous fistula (DAVF) is an abnormal vascular connection between dural arteries and meningeal veins, cortical veins, or venous sinuses. They constitute between 10% and 15% of intracranial arteriovenous malformations [71]. Among the DAVFs, 3.6–4.2% are hypoglossal canal DAVFs, which were attributed to venous hypertension of the hypoglossal canal venous plexus and pulsatile compression [72]. Although the presence of DAVFs in some patients has been found to correlate with previous traumas, craniotomies, or dural sinus thromboses, they are predominantly idiopathic. The typical age of the patient varies from the fifties to the sixties [71]. The symptoms are closely associated with the exact anatomical location of the shunt, although particular attention should be paid to reported pulsatile tinnitus or involuntary tongue movements [71,72,73].

DAVF-induced compression was reported to be present within the hypoglossal canal with its enlargement visible under MRI [73,74]. In reported cases, the arteriovenous fistulas were mostly supplied by the branches of the ECA, with a dominant contribution from the ascending pharyngeal artery. The jugular drainage was provided either by the jugular vein or the interior and exterior venous plexuses [73,74]. Combarros et al. [18] postulate that the HN injury observed in one case had a hemodynamic origin resulting from blood being stolen from the ascending pharyngeal artery to the transverse sinus.

### 2.6. Posterior Inferior Cerebellar Artery

The PICA has a highly variable relationship with the rootlets of the HN, running through (12.5%), above (37.5%), or below them (47.5%). However, all three variants are only possible when the PICA arises from the premedullary part of the VA [75]. The type heading through the rootlets may exert pressure on them and have a direct influence on their stretching, which may be significantly aggravated by the presence of any pathology in the PICA in this area [76,77].

Ekuma et al. [76] reported a case of a fusiform aneurysm at the origin of the PICA from the VA, which was involved in compressing the rootlets of the HN. The aneurysm exhibited pathological adherence to the compressed roots, which likely contributed to the development of the disease.

### 2.7. Occipital Artery

A true aneurysm of the proximal OA is an uncommon finding, and surgical management can pose a greater technical challenge than for these localized in its medial or distal part. Their presence can be linked with connective tissue diseases or neurofibromatosis [78]. However, pseudoaneurysms of the OA are mainly associated with trauma and complications after surgical procedures [79]. Both of them typically manifest as pulsatile masses with coexisting neuralgia, demonstrating symptoms of compression of the surrounding tissues [79]. When these pathologies of the OA occur, they can be involved in HN pinching.

**Figure 4:**
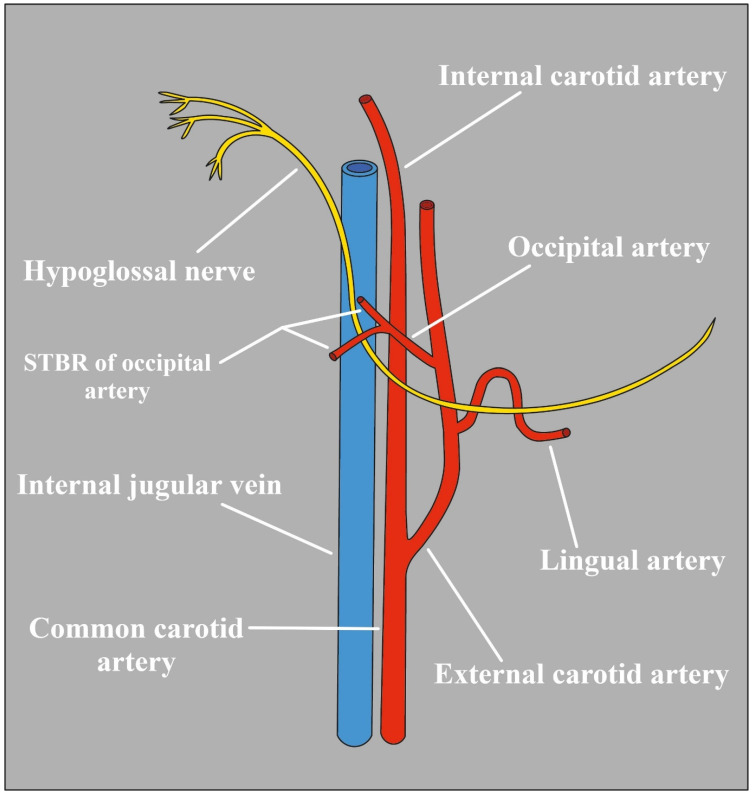
The HN and the adjacent vessels STRB upper and lower sternocleidomastoid branches.

Compression can also occur independently of the changes in the arterial wall, demonstrating that pressure does not necessarily have to be exerted against a firm structure (such as a bone) in order to provoke symptoms. Scotti et al. [80] observed a neuropathy caused by an HN trapped between an ICA with an anomalous tortuous shape and the sternocleidomastoid branch of the OA. However, this double vessel etiology should be considered extremely rare (Figure 4).

## 3. Iatrogenic Factors

It is hard to determine the true frequency of iatrogenic injury among all cases of HN palsy. Stino et al. [13] report that postoperative complications accounted for 29.3% of cases, while an older study by Keane [15] indicates only 5%: an interesting finding, considering that the latter study would use older techniques and hence be more likely to experience complications. These numbers are strongly dependent on the environment of the particular hospital, the available departments, and the characteristics of the patients, so they should not be treated as a definitive answer. Surgery-related complications may be caused by both the direct actions of the surgeons, while compressive neuropathies may be induced by airway management or central venous catheterization performed by the anesthesiology team [13]. Another substantial group includes complications after radiotherapy.

### 3.1. Carotid Endarterectomy

The most common procedure for treating extracranial atherosclerosis is carotid endarterectomy. This procedure involves the surgical removal of the plaque that obstructs the artery. The incidence of the HN injury is reported as 3.79% and is the second most commonly injured nerve in this type of surgery after the vagus nerve. Only 3.96% of these injuries were permanent (i.e., 0.15% of total cranial nerve injuries). The most plausible reasons for the occurrence of neuropraxia are pressure from edema, retraction, and thermal or electrical injury [5,81].

The risk of cranial nerve injury can be increased by bleeding, urgent procedures, and return to the operating room after a neurological incident [81]. Cevik et al. [82] reported that the concurrence of two anatomical variants may aggravate the risk of HN-related complications. The first was the presence of a direct smaller vein near the carotid bifurcation, the second was the intersection of the nerve with the mentioned vein. In these variants, the vein is particularly susceptible to accidental damage during retraction which may result in abrupt bleeding. The subsequent diathermy may contribute to HN injury. Hence, prior ligation is suggested as a preventative measure when the problematic vein is present. The other proposed mechanism is damage to the vessels that supply the nerve along its course and subsequent ischemia [5]. Permanent damage to the HN can result from prolonged stretching or complete transection [83].

Kojima et al. [83] proposed that intraoperative mapping of the HN might be beneficial in reducing the risk of accidental injury in cases where it cannot be precisely identified. According to Ghedia et al. [84], the posterior belly of the digastric muscle is a key landmark for determining HN position in submandibular gland surgeries and level 1 [85] neck dissections. The muscle lies deep and inferior to the submandibular gland and anteriorly to the posterior edge of the mylohyoid muscle. These two muscles create a triangle with the HN acting as the third line. Following either muscle from its intersection allows the identification of the nerve.

### 3.2. Other Surgeries

Cervical spine surgeries taking an anterior approach are considered to be safe and pose a low risk of nerve damage. When such damage happens, it typically influences the recurrent laryngeal nerve, although HN involvement can sometimes occur. Blankenship et al. [86] described a case of dysarthria and dysphagia which occurred immediately after C3–C7 fusion surgery following long-standing complications of the RA. Furthermore, Lee et al. presented concomitant palsy of the HN and the trigeminal nerve lesion after C3–C4 fusion surgery [87].

Such dysphagia might misleadingly imply a more plausible complication from the recurrent laryngeal nerve. This impairment of the HN can occur as a result of the necessity of retracting the ICA, muscles (such as the infrahyoid muscles), and other soft tissues. Such circumstances can lead to secondary compression, traction, and ischemia [88,89]. Although injuries resulting from cervical spine surgeries are mostly reversible and present satisfying outcomes after conservative treatment, permanent damage has been reported [90]. In addition, tumor excisions (including thyroidectomies), tonsillectomies and, in very rare cases, submandibular gland excisions (<1.4%) can also pose a direct risk to the HN [15,91,92]. Also, HN injury can occur in 0.42% of functional neck dissection procedures [93].

The HN can sometimes be linked with dental procedures. Marino et al. [94] describe the case of an 11-year-old child who developed unilateral HN paralysis as a consequence of a mobile orthodontic device application. The patient required one year of speech therapy in order to restore tongue function.

### 3.3. Airway Management

Neuropathy can also be caused by the use of a mask or orotracheal tube. Shaha et al. report that of all studied cases of HN palsy after general anesthesia, 66.7% were limited to the HN, 21.7% were combined with the recurrent laryngeal nerve, and 11.6% with the lingual nerve [95].

#### 3.3.1. Laryngeal Mask Airway

Although there have been many reports of HN palsy following the use of laryngeal mask airway (LMA), it should still be classified as a rare complication. This is believed to result from the compression of the HN against the greater horn of the hyoid bone as the HN enters the floor of the mouth [96]. In a correctly placed mask, the nerve lies above the hyoid bone on the anterolateral aspect of the upper end of the mask [96]. The onset of symptoms may occur within the first couple of hours after the procedure or be delayed until the next day [97,98]. In the case by Takahoko et al. [99], the nerve paralysis appeared the day following the surgery but was not observed initially in postoperative assessment after the mask was removed.

Although LMA-induced neuropathy is expected to be unilateral, the bilateral cases were also described [97,100].

The events were described among a few types of LMAs: Supreme [99], ProSeal [98], and Classic [97,100,101,102,103,104,105]. This has been attributed to extreme head-side rotation over a long period of time, the use of nitrous oxide during anesthesia, which diffuses into the cuff and increases its pressure, and inadequate size and malposition of the LMA [97,100]. This complication has been reported despite following the procedures and using the correct volume of air inserted into the cuff; however, to avoid similar complications, it has been proposed to avoid the maximal recommended pressures as this would not compromise mask function [98].

The condition is expected to undergo complete self-resolution: it occurs within two months after the procedure in 50% of patients and within four months in 80%. As such, any aggressive treatment should only be performed after careful consideration [95]. The recovery period is expected to be six months, and hence the follow-up should not be scheduled within this time [99].

In some cases, iatrogenic injuries associated with airway management may also result in neuropathy to the lingual nerve. The lingual nerve is a branch of the trigeminal nerve arising from its mandibular division. Any damage to this nerve results in numbness and impairment in taste sensation [106].

Ulusoy et al. [107] propose five factors (next to laryngoscopy) that can contribute to anesthesia-induced neuropraxia of the HN and the lingual nerve. These include mask compression and the triple maneuver, which can result in prolonged compression against the mandible angle, the presence of an oropharyngeal airway while performing a maneuver, cricoid pressure during laryngoscopy, and tight attachment of an endotracheal tube; also, the properties of the endotracheal tube (e.g., high pressure, no flexibility, and relocation) can also have a negative role. While each individual factor may have a limited impact on nerve performance, their effect intensifies when acting simultaneously.

The causes of lingual nerve neuropathies are reported to be similar to isolated HN neuropathies following LMAs. Although many more predisposing factors have been proposed, such as the use of anticoagulants or lidocaine lubrication, they have only been noted in singular cases and should be treated with caution [108].

#### 3.3.2. Intubation

General anesthesia, and subsequent intubation, are believed to play a role in HN palsy. In many cases, the mechanism is similar to that associated with airway management with a mask. Such injury might also be provoked by direct compression of the hypopharynx region by the posterior part of the laryngoscope or orotracheal tube, resulting in bruising and subsequent injury. It may also be secondary to tongue swelling caused by prolonged surgical procedures. In such cases, major fluid shifts may occur together with compression of the nerve between the endotracheal tube and the hyoid bone [104,109,110]. Additional factors might be stretching of the nerve on the anterior aspect of the transverse process of the C1 vertebra, which is provoked by neck hyperextension or compression between the laryngoscope blade and the pathologically calcified stylohyoid ligament. Both direct and suspension laryngoscopy can affect the HN [90,95,111].

Prolonged intubation can also be a problem in intensive care units, as noted during the COVID-19 pandemic. In a study by Decavel et al. [112], 11% of patients who underwent mechanical ventilation in the prone position developed palsy of at least one cranial nerve, and 90% of these patients had neuropathy of the HN.

It is worth noting that both the implementation of the endotracheal tube and its removal increase the risk of injury. Extubation with a dilated cuff was mentioned as a cause of HN neuropraxia by exerting pressure on the nerve against the greater horn of the hyoid bone. Therefore, caution is advised whenever external clearance of the airways is necessary, especially in patients who require recurrent airway management, such as those with extensive trauma [110,113,114]. 

Iatrogenic injuries of the HN can also occur outside the surgery. For example, prolonged usage of orthosis in a patient with a weakened cervical muscle resulting from mitochondriopathy was found to result in severe unilateral wasting and weakness of the tongue [115].

### 3.4. Tapia’s Syndrome

Tapia’s syndrome (TP) is a concomitant deficit of the HN and the recurrent laryngeal branch of the vagus nerve as a consequence of extracranial injury [116]. The typical symptoms consist of a loss of motor function typical of HN damage, together with ipsilateral vocal cord paralysis. TP can occur both unilaterally and bilaterally. Full or partial resolution is expected within six months after the onset of symptoms; this implies that the injury is neuropraxic and results from compression [109]. It is recommended that airway endoscopy be used for assessing vocal cords [117]. Although TP is mainly associated with surgical procedures and/or airway management, including nasotracheal intubation, incidents have also been reported following infective agents, vascular complications, and tumors [118,119].

The typical patient with TP is young and male [118]. TP seems not to be associated with any specific type of intervention since it has been reported in a range of surgeries including liver transplantation, aortic valve replacement with bypass grafting, rhinoplasty, arthroscopic shoulder surgery, plastic surgery, and spine surgery [109,116,118,119]. However, a common factor seems to be the position of the patient and its change during the procedure (from supine to prone or sitting position) [109,116]. The compression of the recurrent laryngeal branch of the vagus nerve might result from compression against the posterior part of the thyroid cartilage or mandible [119,120].

Therefore, when neurological symptoms occur after airway management related to inter alia intubation, they should not be exclusively associated with injuries strictly limited to the HN. In addition to symptoms provoked by neuropraxia of the HN, intubation can also result in a range of complications with a non-neural background, such as sore throat, pharyngeal dryness, respiratory difficulties, and hoarseness caused by damage to the pharyngeal nerves [107].

The proposed treatment consists of vitamins B1, B12, and alpha lipoic acid. Acute cases can also be treated with glucocorticosteroids (systemic or local) and injections of hyaluronic acid [116,118]. Another recommended form of treatment is speech therapy. If a satisfying outcome is not achieved, an additional vocal cord gel implant might be injected [117,119].

Currently, recognition of the TP as a separate entity has mostly historic significance, with little application to clinical practice. Similar doubts apply to other cranial nerve neuropathies with eponymic names i.e., Collet–Sicard, Villaret’s, and Jackon’s syndromes [121,122]. A combination of deficits in the neighboring nerves should be rather diagnosed based on symptoms and treated accordingly. The extent of nerve damage in TP is also unclear, as some authors also describe symptoms from the accessory nerve (weakness of trapezoid and sternocleidomastoid muscles) and the cervical sympathetic trunk, resulting in Horner’s syndrome [120].

### 3.5. Radiotherapy

Not all cases of iatrogenic HN dysfunction are related to surgery or result from some kind of kinetic influence. It has been found that 5.1% of patients suffer from cranial nerve dysfunction after radiotherapy. The HN is believed to suffer the highest complication rates among them [123,124].

A study with a median eight-year follow-up found that 8.7% of patients suffered from radiotherapy-induced HN palsies secondary to the treatment of nasopharyngeal carcinoma. Of these, 74% had unilateral dysfunction, and in 1.3%, the dysfunction was noticed as secondary to tumor recurrence. It is important to note that the latency period between the treatment and the onset of symptoms is over six years. Chemotherapy was shown to increase the chance of symptoms [123,125]. When this condition occurs after high-dose radiotherapy, it demonstrates resistance to treatment such as steroids, pentoxifylline–tocopherol–clodronate, and hyperbaric oxygen, and should be considered permanent [123]. The proposed mechanism is microvascular injury resulting in ischemia which aggravates the pre-existing vascular problems [126,127].

## 4. Pathologic Masses

### 4.1. Osteophytes

**Figure 5:**
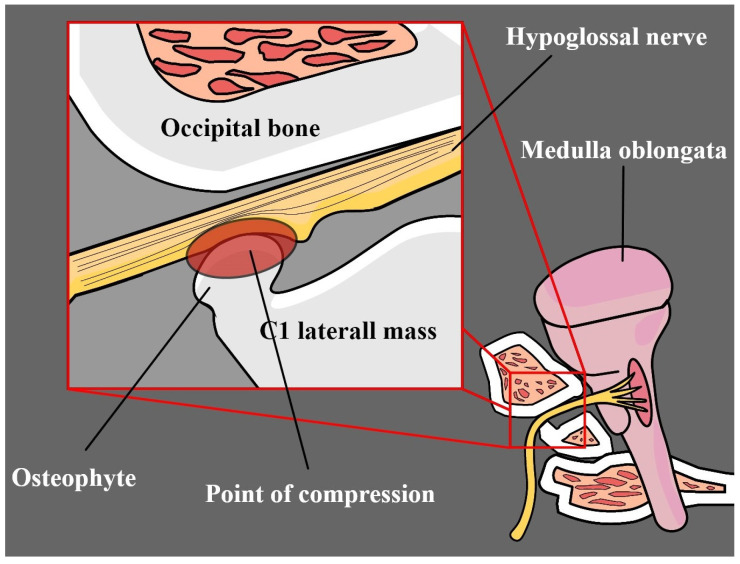
Compression of the HN by an osteophyte based on Patro et al. [130].

One of the most unusual causes of HN neuropathy is compression induced by osteophytes at the exit from the hypoglossal canal. Ozaki et al. [128] describe the case of a patient who suffered from atlantoaxial dislocation with osteophyte involvement with concomitant rheumatoid arthritis. In a similar case, an osteophyte was found to arise from the atlantoaxial joint and induce neurological symptoms related to the HN (Figure 5) [129,130]. Although symptomatic osteophytes are not uncommon, they rarely affect the HN. It is recommended to use both MRI and high-resolution CT in diagnostic imaging [130].

### 4.2. Cysts

Intraneural ganglion cysts are typically found in the peripheral nervous system, near the tendon sheaths or joints. It is believed they might arise as a result of capsular defects caused by trauma, metaplasia, developmental rests, or repetitive stress [131,132]. Migration of substances from the joint contributes to the growth of the lesion and myxoid degeneration [133,134]. The nerve can also be affected by arachnoid and juxtafacet cysts from the atlantooccipital joint [133,135,136]. Mujic et al. [135] propose that synovial cysts may demonstrate significant growth spanning from the joint up to nerve rootlets and the medulla oblongata. The cysts can become symptomatic when they compress the nerves or the spinal cord following overgrowth, acute hemorrhage, or tissue invasion, but they typically remain unobtrusive [132,137]. The presence of a cyst inside the hypoglossal canal can be problematic and elevate the risk of complications when taking samples for biopsy. As it is possible to encounter intradural, juxtafacet, and arachnoid cysts in this area, imaging plays a vital role in the diagnostic process [138,139,140].

The presence of a cyst may manifest as pain and neck discomfort. The differential diagnosis should include more common entities such as hypoglossal schwannoma or epidermoid tumors [141].

Intracranial arachnoid cysts are commonly found in the posterior cranial fossa, middle cranial fossa, and the sellar-suprasellar region. In some instances, they reach from the posterior cranial fossa to the hypoglossal canal; this can cause compression of the nerve and sometimes the medulla oblongata [142,143]. The prevalence of such cysts in the general population is estimated at 2.3% [144]. The suspected etiology is inflammatory, traumatic, or congenital but is mostly unknown [142,145].

In symptomatic cases, surgery should be considered, especially when the medulla oblongata is also affected by the presence of pathologic masses [137]. Such treatment of arachnoid cysts was commonly based on paramedian suboccipital craniotomy with inner entrance to the hypoglossal canal; however, Burkhardt et al. [140] demonstrated that the direct transcondylar approach is also a reliable technique.

### 4.3. Tumors

The most common cause of HN palsy is the presence of a tumor, which can be expected at any point along its length [2,25]. In locations with very limited space, such as the hypoglossal canal, the nerve becomes particularly susceptible to any external pressure from tumors originating within or extending toward the canal. This predisposes to the early onset of symptoms [146]. Hypoglossal canal and jugular foramen tumors are estimated to represent 0.3% of all intracranial tumors [147]. The most common tumors causing HN include paraganglioma, lymphoma, schwannoma, chordoma, nasopharyngeal carcinoma, meningioma, or distant metastasis [3,13,15]. At the cisternal and canalar levels, the most frequent tumors are hematogenous skull base metastases [2]. A prior history of previous cancer diseases is recognized as a predisposition to HN palsy [13].

### 4.4. Collet–Sicard Syndrome

Collet–Sicard syndrome (CS) is paralysis of the cranial nerves from IX to XII as a result of penetrating or blunt trauma, tumors, inflammation, abnormalities in the craniocervical junction, and vascular lesions [148,149]. Its most common cause is believed to be a tumor of the ear [150]. The vascular etiology includes ICA coiling, dissection or pseudoaneurysm, and IJG or sigmoid sinus thrombosis [121,150,151,152,153]. It is worth noting that CS is the most common neurologic complication of Jefferson fracture: a burst type of C1 vertebra fracture [149]. However, most Jefferson fractures are not associated with any cranial nerve disorders [148].

The constellation of symptoms from a large number of cranial nerves may suggest that the ongoing pathology is localized focally in the intercondylar space, i.e., close to the jugular foramen and the hypoglossal canal [121].

A similar syndrome to CS is Villaret’s syndrome, characterized by paralysis of the cranial nerves from IX to XII with Horner’s syndrome, and Jackon’s syndrome, presenting as involvement of the cranial nerves from X to XII [150].

### 4.5. Godtfredsen Syndrome

Godtfredsen syndrome (also called clival syndrome) is a concomitant pathology of both the abducens nerve and HN. Its presence could be a prognosis of underlying focal pathology localized at the clivus, but cases with pathologic changes in the subarachnoid space or medulla oblongata were also observed [154]. Its most typical cause is either a local tumor or distant metastases [155]. However, due to the limited number of cases, it is not possible to definitely establish the most plausible type of malignancy. Other reported causes include meningitis, mastoiditis, hematoma, or self-limiting neuropathy [154].

## 5. Trauma

Although the HN is generally resistant to the effects of blunt trauma by virtue of its location, it is not protected from piercing damage, such as gunshots [156]. It is not easy to estimate the prevalence of the latter since it is strictly dependent on the social and legal aspects of the patient’s environment. Overall traumatic etiology accounts for around 4% of total cases of HN palsy [13]. However, it has been noted that the traumatic incident may not display any abnormalities in diagnostic imaging [30,157]. The most typical traumatic injury is caused by a fracture within the base of the skull in the hypoglossal canal or by damage to the cervical vertebrae and traction of the nerve such as in an odontoid fracture. In very rare cases, disorders of the spinal cord or the medulla oblongata can also be involved in HN paralysis. The incidence of syringomyelia ranges from 0.3% to 3.2% of spinal cord injuries. Post-traumatic syringomyelia leading to secondary syringobulbia has been reported by Mousele et al. [158,159]. Syringobulbia may occur independently or as a result of tumors, malformations, trauma, or inflammation.

### Occipital Condyle

Occipital condylar fracture (OCF) accounts for 4% to 19% of cases of patients with craniospinal traumatization and 0.4% to 0.7% of severely traumatized patients [160]. The major cause is high-energy trauma, possibly with severe head injury [161]. The onset may be immediate, or occur two days to nine weeks after the initial trauma [162]. OCFs can be divided into three subtypes according to the Anderson and Montesano classification: Type I—comminuted fracture, Type II—extension of fracture of the skull base, and Type III—avulsion fracture of the condyle with alar ligament involvement [163]. All types can be treated conservatively if there is no ligamentous instability or concomitant injury of the neck. Type I and II are typically treated with a cervical collar. Halo fixation can be taken into consideration in Type III [161]. However, in most patients, the condition does not significantly improve over time [164].

It is hard to estimate the true incidence of cranial nerve deficits after OCF. This might be in part a consequence of the significant portion of false-negative patients in whom conventional CT scans and radiography did not detect the fraction. Hence, additional electrodiagnostic studies and high-resolution CT should be considered for more detailed evaluation [165]. According to Vadivelu et al. [164], delayed cranial nerve palsy occurs in less than 1% of OCF cases. However, Tuli et al. [166] report that 31% of patients had symptoms of lower cranial nerve injury and 38% of them had a delayed onset. The clinical manifestation is very diverse as it includes a wide range of symptoms ranging from alteration of consciousness, occipital pain, and neural deficits to a complete lack of symptoms.

The proposed etiologies include stretching the nerve at the exit of the canal caused by callus formation during healing, impingement by bone fragments in the proximity of the canal, fracture instability, and edema. In addition, traumatic events such as traffic accidents can also give rise to complications associated with intubation [164,166]. Occipital condyle syndrome is generally a symptom of bone metastasis and can be one of the first symptoms of underlying malignancy. Even small space-occupying lesions can provoke disproportionally intense pain symptoms and HN palsy. Hence, such patterns of symptoms should not be neglected [164].

Occipital condyle syndrome is indicated by the presence of ipsilateral palsy of the HN and unilateral occipital pain. In such cases, the pain worsens with contralateral rotation of the head, radiates towards the mastoid, vertex, and ear, and is associated with tenderness of the scalp [56].

## 6. Systemic Diseases, Infections, and Inflammations

It is estimated that 4% to 7.3% of HN palsies are of viral or bacterial origin [13,15]. In such cases, neuropathy presumably occurs as a result of either the direct influence of the agents on the nerve structures or the immunological response against them [167]. Some cases do not resolve immediately after the end of infection. Hadjikoutis et al. [168] reported a case of postinfectious (streptococcal) dysfunction of the HN, which partially improved three months after the onset of symptoms.

However, not all cases of such palsies can be explained by immunological interactions. In some instances, the neuropathy is a result of edema of affected tissues: inflammation in the oral cavity, such as an infected molar, can provoke HN dysfunction secondary to the expansive swelling [169]. Such swelling-induced neuropathy may also occur after tooth extraction due to the injections and tissue trauma aggravating edema [170]. In addition, the HN has also been reported to be compressed by enlargement of the reactive deep cervical lymph nodes following bacterial infection [167].

Interestingly, temporary HN palsy was reported as a complication after vaccine administration, such as the anti-SARS-CoV-2 vaccine, and in combination with mononeuritis multiplex treatment [171,172].

Other cases have been associated with tonsillitis or Ebstein–Barr virus, and autoimmunologic diseases such as Wegener’s granulomatosis, multiple sclerosis, and Guillain–Barré syndrome [15,25,173,174]. Studies have also reported the palsy to result from encephalomyelitis and from the thickening of the dura mater in the hypoglossal canal in hypertrophic pachymeningitis [174,175].

### 6.1. Rheumatoid Arthritis

Cervical spine involvement may be noted in up to 86% of patients suffering from rheumatoid arthritis; of these, 65% have atlantoaxial subluxation, mostly anterior, and 15-20% have vertical subluxation [176]. The erosion of the odontoid and atlantooccipital joint and damage to the apical and the alar ligaments enable vertical movement of the odontoid towards the foramen magnum. This results in compression of the cranial nerves, including the HN, close to the transverse process of the atlas [86]. In more severe subluxations, such compression may occur near the entrance of the hypoglossal canal [86]. Both bilateral and unilateral cases have been reported [177,178], and damage may involve both the glossopharyngeal and vagus nerves [179]. However, most patients have no signs of HN dysfunction despite radiological evidence of spine involvement [86].

Another possible mechanism of HN neuropathy in patients with rheumatoid arthritis is compression by an inflammatory pannus arising from the atlantoaxial joint and extending toward the hypoglossal canal. However, this cannot be considered a common finding [86,180].

### 6.2. Tuberculosis

It has been estimated that 0.3–1% of spinal tuberculosis cases, also known as Pott’s spine, can be classed as tuberculosis of the craniovertebral junction [181]. It is important to note that this rare form is characterized by C1–C2 vertebrae presentation along with cranial nerve palsy. It can progress without any systemic symptoms and typically affects more than just the HN [182]. Damage to the HN may be provoked by two mechanisms. An abscess in the area of the prevertebral fascia (the membrane extending from the base of the skull to the superior mediastinum) leads to direct compression of the nerve axons. Atlantooccipital dislocations and subluxations reflect the damage to the ligamentous apparatus occurring during the typical course of spinal tuberculous. These injuries result in impingement of the HN within the canal [183]. The presence of the disease in this region mainly comes from the retropharyngeal area, with few cases associated with the primal bone origin [183].

The onset of symptoms may be insidious, manifesting as head or neck pain and neck stiffness. This variant might be encountered in both adults and children [184]. However, it should be emphasized that tuberculosis-induced palsy of the HN does not necessarily need to be linked with its spinal variant. Thakur et al. [185] describe a case of a tubercular lesion located on the posterior wall of the nasopharynx without any bone erosion. The proposed explanation was a minimal invasion into the nerve or meningeal membranes. Other manifestations affecting the HN include tubercular meningitidis and adenopathy (Table 3) [185].

## 7. Conclusions

HN neuropathies are not common entities. Sometimes their diagnosis might be complicated due to a lack of clear imaging, laboratory findings, and the high variety of possible etiologic factors. For that reason, it is important to consider the typical clinical image with the pattern of symptoms related to the level of the lesion. Although the most common cause of HN neuropathy is cancer, other causes, including iatrogenic ones, should not be disregarded, as many complications can be avoided with appropriate caution. It is important to remember that concomitant impairment of adjacent nerves can drastically alter the diagnostic process and treatment outcome. This topic requires more comprehensive clinical studies, especially involving paralyzes of vascular etiology, which would standardize the management guidelines present so far only in case reports. A thorough knowledge of the anatomical intricacies associated with the condition is necessary to allow proper evaluation of such cases and appropriate treatment.

## Figures and Tables

**Table 1:** Branches of the hypoglossal nerve.

	Branches of the Hypoglossal Nerve				
	Meningeal	Descending	Thyrohyoid	Geniohyoid	Muscular
Supplied structures	Diploë of the occipital bone Dural walls of the occipital and inferior petrosal sinuses Floor of the posterior cranial fossa’s anterior wall	Omohyoid Sternohyoid Sternothyroid	Thyrohyoid muscle	Geniohyoid muscle	Intrinsic tongue muscles: Longitudinal superior Longitudinal inferior Transverse Vertical Extrinsic tongue muscles: Genioglossus Hyoglossus Styloglossus Chondroglossus
Point of origin	C1/C2 nerves	C1/C3 nerves	C1 nerve	C1 nerve	Medulla oblongata

**Table 2:** Segments of the hypoglossal nerve distal to its nuclei at the medulla oblongata—combined classifications according to Iaconetta et al. and Thompson and Smoker [2,8].

	Segments of the Hypoglossal Nerve				
				Sublingual	
	Cisternal	Canalar/Skull Base	Descending/ Carotid Space	Horizontal	Ascending
Adjacent Structures	Medulla oblongata Posterior inferior cerebellar artery VA Cranial nerves: IX X XII	Hypoglossal canal: Inferiorly: occipital condyle Laterally: jugular foramen, jugular process of the occipital bone Supero-medially: sphenoid part of clivus	Sternocleidomastoid muscle Stylian muscles ICA Cranial nerves: IX X XII	Greater horn of hyoid bone Submandibular gland Subcutaneous fat Lingual artery Muscles: Middle pharynx constrictor Hyoglossus Stylohyoid	Mylohyoid muscle Tongue Lingual artery Wharton duct
Distal boundaries	Dural pores in the posterior fossa	Exit of the neurocranium in the nasopharyngeal carotid space	Anterior margin of the sternocleidomastoid muscle	Edge of mylohyoid muscle, at the inferior surface of the tongue	Two to five terminal branches to the muscle bellies

**Table 3:** Causes of the HN palsy.

Groups of Etiologies	Cause of Palsy
Vessels	Pathological changes or abnormal morphology of: Internal carotid artery External carotid artery Vertebral artery Occipital artery Posterior inferior cerebellar artery Abnormal vessels: Dural arteriovenous fistulas Persistent vessels
Iatrogenic factors	Airway management
	Radiotherapy
	Intraoperative injuries
Pathologic masses	Tumors
	Cysts
	Osteophytes
Trauma	Direct damage
	Occipital condyle fractures
	Atlantoaxial subluxation (also secondary to the rheumatoid arthritis or tuberculosis)
Immunologic factors	Autoimmunologic diseases
	Infections
	Vaccinations
	Oral cavity inflammations

## Abbreviations

HN	hypoglossal nerve
VA	vertebral artery
ICA	internal carotid artery
ECA	external carotid artery
IJG	internal jugular vein
C	cervical spinal nerve/cervical vertebra
OA	occipital artery
PICA	posterior inferior cerebellar artery
DAVF	dural arteriovenous fistula
LMA	laryngeal mask airway
TP	Tapia’s syndrome
CS	Collet–Sicard syndrome
OCF	occipital condyle fracture
